# Changes in Physical Activity Pre-, During and Post-lockdown COVID-19 Restrictions in New Zealand and the Explanatory Role of Daily Hassles

**DOI:** 10.3389/fpsyg.2021.642954

**Published:** 2021-02-25

**Authors:** Elaine A. Hargreaves, Craig Lee, Matthew Jenkins, Jessica R. Calverley, Ken Hodge, Susan Houge Mackenzie

**Affiliations:** ^1^School of Physical Education, Sport & Exercise Sciences, University of Otago, Dunedin, New Zealand; ^2^Department of Tourism, University of Otago, Dunedin, New Zealand; ^3^Department of Psychological Medicine, University of Otago, Wellington, New Zealand

**Keywords:** behavior change, exercise, psychology, stress, physical activity intensity, COVID-19

## Abstract

Covid-19 lockdown restrictions constitute a population-wide “life-change event” disrupting normal daily routines. It was proposed that as a result of these lockdown restrictions, physical activity levels would likely decline. However, it could also be argued that lifestyle disruption may result in the formation of increased physical activity habits. Using a longitudinal design, the purpose of this study was to investigate changes in physical activity of different intensities, across individuals who differed in activity levels prior to lockdown restrictions being imposed, and across three time periods: pre-, during- and post-lockdown. This study also examined the extent to which the experience of daily hassles explained any changes in physical activity. A convenience sample (*N* = 759) recruited through social media, provided data from an online survey administered during weeks 2–3 of a 5-week lockdown and 231 participants provided complete data again 6 weeks post-lockdown (72% female, *M* age = 43 years). Participants completed the International Physical Activity Questionnaire–Short Form and the Daily Hassles Scale. Results showed that vigorous and moderate intensity PA were significantly lower during- and post-lockdown compared to pre-lockdown in those individuals who had been highly active pre-lockdown. In contrast, for moderately active individuals pre-lockdown, vigorous and moderate intensity PA was significantly higher during-lockdown compared to pre-lockdown, and these increased levels of vigorous PA were maintained post-lockdown. Participants experienced daily hassles due to inner concerns, time pressures, family, and financial concerns to the same extent during- and post-lockdown. Those daily hassles had a small negative (Standardized β = −0.11; *p* < 0.05) predictive effect on post-lockdown PA. It appears that to understand the effect of COVID-19 restrictions on PA, the activity status of individuals pre-lockdown needs to be taken into account. The daily hassles appeared to play a role in post-lockdown PA behavior, but future research should investigate why these results occurred.

## Introduction

In March 2020, the New Zealand (NZ) Government instigated Level 4 lockdown restrictions in response to the World Health Organization (WHO) declared COVID-19 pandemic (World Health Organisation, 2020). These Level 4 lockdown restrictions (henceforth termed “lockdown”) urged all New Zealanders (except those classed as essential workers) to stay at home unless undertaking a limited range of “essential” activities (e.g., shopping for groceries, medical reasons) (New Zealand Government, 2020a). Importantly, the NZ government allowed individuals to be physically active in their local neighborhood, imparting to the NZ public the importance of being physically active for health and well-being during this period (New Zealand Government, 2020b). The physiological and psychological benefits (e.g., reductions in depression and anxiety levels) of being physically active are well-recognized (World Health Organization, 2010), and more specific benefits related to the effects of COVID-19 have been proposed (e.g., Matias et al., 2020; Simpson and Katsanis, 2020; Woods et al., 2020). In particular, the COVID-19 pandemic and its associated restrictions have resulted in substantial psychosocial effects, for example increased prevalence of anxiety and stress (Salari et al., 2020), which physical activity (PA) is known to ameliorate (Stubbs et al., 2017). Consequently, researchers have opinionated on the importance of being physically active during the pandemic (Chen et al., 2020; Hudson and Sprow, 2020; Lippi et al., 2020; Ricci et al., 2020; Sallis and Pratt, 2020).

The NZ lockdown constituted a significant lifestyle change for individuals. Many people were either not working or were working from home, plus schools, indoor exercise and recreation facilities were closed, club/community sport was canceled, and outdoor recreation was limited to local neighborhoods. These unprecedented conditions constituted a population-wide “life-change event,” defined by the US National Library of Medicine as “those occurrences, including social, psychological and environmental, which require an adjustment or effect a change in an individual's pattern of living” (Engberg et al., 2012, p. 433). Life-change events disrupt a person's daily routine and are a known determinant of physical activity (PA) behavior change (Engberg et al., 2012). This particular life-change event may have facilitated old habits being broken (e.g., patterns of inactivity) and the formation of new habits (e.g., adoption of PA), in part because time-related barriers for PA may be removed. Alternatively, the lockdown restrictions may have decreased normal physical activity levels (e.g., due to exercise facilities being closed, or recreational areas being unavailable) or made people less likely to be physical active (e.g., due to increased childcare demands, or COVID-related anxiety). Consequently, investigating how PA changed in the NZ context as a result of the lockdown provides valuable insights on the ramifications for health-related behavior.

At the outset of the COVID-19 pandemic, researchers suggested PA levels would decline (e.g., Papaioannou et al., 2020). In 2020, studies from different countries (e.g., Belgium, Canada, Greece, USA, Australia) investigated changes in PA behavior as a result of their varied COVID-19 restrictions. Most of these studies used cross-sectional designs with online survey methods, collecting data at one point during lockdown along with retrospective assessment of PA prior to COVID-19 restrictions being implemented. PA behavior was assessed in different ways; for example, with validated self-report measures, non-validated measures or a single item question asking whether PA changed. Summarizing this research, PA was shown to be lower during COVID-19 restrictions compared to before restrictions were put in place (López-Bueno et al., 2020; Mutz and Gerke, 2020; Schnitzer et al., 2020; Stanton et al., 2020). More nuanced analysis has shown that between 24 and 49% of samples had decreased PA levels during restrictions, 21–32% had increased PA levels, and 30.5–44% had no change in PA (Brand et al., 2020; Knell et al., 2020; Mutz and Gerke, 2020; Stanton et al., 2020).

Other research has shown that changes in PA differed as a function of individuals' pre-lockdown PA levels. Meyer et al. (2020) found 32% of their US sample who were active pre-COVID restrictions decreased their PA levels during restrictions, while PA levels remained unchanged for those who were inactive prior to restrictions. Barkley et al. (2020) used tertile splits based on PA scores pre-COVID restrictions from the Godin Leisure-Time Exercise Questionnaire (Godin and Shephard, 1985) to create, low, moderate and high active groups. They found that US university staff and students classified as high active (average of 75.8 METs/week) reduced their PA during the COVID-19 restrictions by 22%; those classed as moderately active (average of 32.5 METs/week) increased their PA by 14%; and those classed as least active (12.9 METs/week) increased their PA by 83%. Also using the Godin questionnaire, Lesser and Nienhuis (2020) found, of the 63% of Canadians classed as inactive pre-COVID restrictions (participating in <150 min/week of moderate to vigorous PA), 40.5% became less active, and 33% became more active during the restrictions. Of the 37% classed as active pre-COVID restrictions (participating in ≥ 150 min/week of moderate to vigorous PA), 22.4% became less active and 40.3% became more active. In Belgium, Constandt et al. (2020) reported that, amongst people classed as “high active” (exercising regularly/at least once a week) prior to COVID-19 restrictions, 36% exercised more, 23% exercised less, and 41% exercised the same during restrictions. Amongst “low active” individuals (exercised non-regularly/less than once a week), 58% exercised more, 7% exercised less, 5% exercised the same, and 30% did not exercise at all.

Given these mixed findings, further investigation of PA behavior in relation to COVID-19 restrictions is warranted, particularly with the use of a validated self-report measure of PA that has been missing in some studies (e.g., Constandt et al., 2020; Meyer et al., 2020; Mutz and Gerke, 2020) and a classification of activity level that corresponds with the WHO PA guidelines. Additionally, research has yet to investigate potential changes in physical activity intensity during COVID-19 restrictions (e.g., vigorous, moderate, walking) and how PA may have changed once lockdown restrictions were eased. NZ provides a unique context in which to study PA after COVID-19 restrictions were lifted because of its success in largely eliminating community transmission which allowed people to return to pre-COVID mobility levels (Wilson et al., 2020).

Research is yet to provide psychological-based explanations for why PA changes have occurred during COVID-19 restrictions (Papaioannou et al., 2020; Sallis et al., 2020). Systematic reviews have shown that, in general, life changes/events have a negative effect on PA participation (Allender et al., 2008; Engberg et al., 2012; Stults-Kolehmainen and Sinha, 2014). However, different life events can have differential effects on PA, with some prompting PA increases (e.g., change in employment status) and others prompting PA decreases (e.g., transition to University, having a child) (see Engberg et al., 2012 for review). Researchers have suggested that, because not everyone responds to life events in the same way, it may not be the particular life event that influences behavior change *per se*, but rather the daily hassles (i.e., stressors) that the event creates in a person's life (Kanner et al., 1981; O'Connor et al., 2009; Uijtdewilligen et al., 2014). For example, DeLongis et al. (1982) showed that a larger percentage of variance in health status was explained by the daily hassles in people's lives resulting from a major life event, rather than stress due to the event itself. Therefore, it may be that the differential effects of COVID-19 restrictions on PA hinges upon the experienced daily hassles created by the event. Indeed, Cheval et al. (2020) suggested a measure of stress should be included in research to examine how it moderates PA change.

Daily hassles have been operationalized as a measure of the everyday stressors (i.e., problems or difficulties that are part of everyday life) that a particular situation causes (O'Connor et al., 2009). The extent of daily hassles has been associated with poorer eating behaviors (O'Connor et al., 2008), lower physical activity (Twisk et al., 1999; Nguyen-Michel et al., 2006) and is predictive of actual stress experienced (Feizi et al., 2012). Nguyen-Michel et al. (2006) found support for their hypothesis that PA would be associated with lower perceived daily hassles, but identified that it was equally plausible that the experience of daily hassles would discourage physical activity participation. In their review of the PA and stress research, Stults-Kolehmainen and Sinha (2014) also found evidence for these bi-directional relationships. Physical activity status may also protect people from experiencing stress as a result of the daily hassles experienced (Feizi et al., 2012). Thus, how active individuals are may influence their response to the daily hassles caused by the COVID-19 restrictions and provide an explanation for PA changes.

The purposes of this study were 2-fold. Firstly, we examined changes in PA from pre-lockdown to PA during-lockdown, and post-lockdown. Specifically, we aimed to examine changes in total PA and PA of different intensities, and compared the PA of individuals who met the WHO PA guidelines pre-lockdown (deemed moderately active), exceeded the PA guidelines (deemed highly active) and who did not meet the guidelines (deemed inactive). We made tentative hypotheses that overall physical activity would decrease during lockdown compared to pre-lockdown, but recover to pre-lockdown levels post-lockdown. We expected highly and moderately active individuals to remain active during- and post-lockdown, and for inactive individuals to increase their PA during- and post-lockdown. The analyses on the changes to PA of different intensities were exploratory. Secondly, we investigated the experience of daily hassles during- and post-lockdown and the extent to which those daily hassles predicted PA during- and post-lockdown. We hypothesized that the experience of daily hassles would be higher during- compared to post-lockdown. We expected that daily hassles would negatively predict PA behavior, but this result would be moderated by the individuals' pre-lockdown PA status.

## Materials and Methods

### Participants and Procedures

A convenience sample of the NZ population was recruited using social media recruitment methods (King et al., 2014) and a virtual snowball recruitment technique (Baltar and Brunet, 2012). Study information with a link to an online survey (hosted on the online survey platform Qualtrics) was shared via Facebook and circulated via email to contacts at NZ universities and other organizations (e.g., regional and national sports organizations, city councils) for dissemination through their networks. The study advertising specifically stated that we wanted to hear from people who were not overly active as well as those who were active in an effort to reduce response bias. Participants were eligible if they were aged 18 years or older and living in NZ for the duration of the lockdown restrictions. Those with any contraindications (e.g., illness, injury) that prevented being physically active during the lockdown restrictions were excluded. These criteria were assessed via screening questions at the beginning of the online survey.

Participants completed the survey at two time points. The first was completed between the second and third weeks of NZ's 5-week lockdown period (April 8 to April 15, 2020; the survey was open for 8 days). We deemed this data point “during-lockdown”. Secondly, those participants who completed the first survey and agreed to be contacted again were emailed the link to the follow-up survey. The follow-up survey was sent on June 9th 2020 and was open for completion for 9 days. Participants were sent a reminder 6 days after the first email was sent. This period was 6 weeks after lockdown had ended and was the final week of four where NZ was subject to Level 2 restrictions. Under these restrictions, people could leave home and travel freely, and were asked to adhere to public health measures (e.g., physical distancing, mask wearing on public transport). All gyms, health clubs and swimming pools were open with public health measures in place. Community sport was also allowed but restricted to a maximum of 100 people in a confined space. We deemed this point “post-lockdown”. Ethical approval was obtained from the university ethics committee (reference: D20/214). All participants provided online informed consent before completing the survey. This study comprises part of a larger project investigating determinants and outcomes of PA as a result of the COVID-19 lockdown restrictions. The full project survey took an average of 12 min to complete.

**Figure 1:**
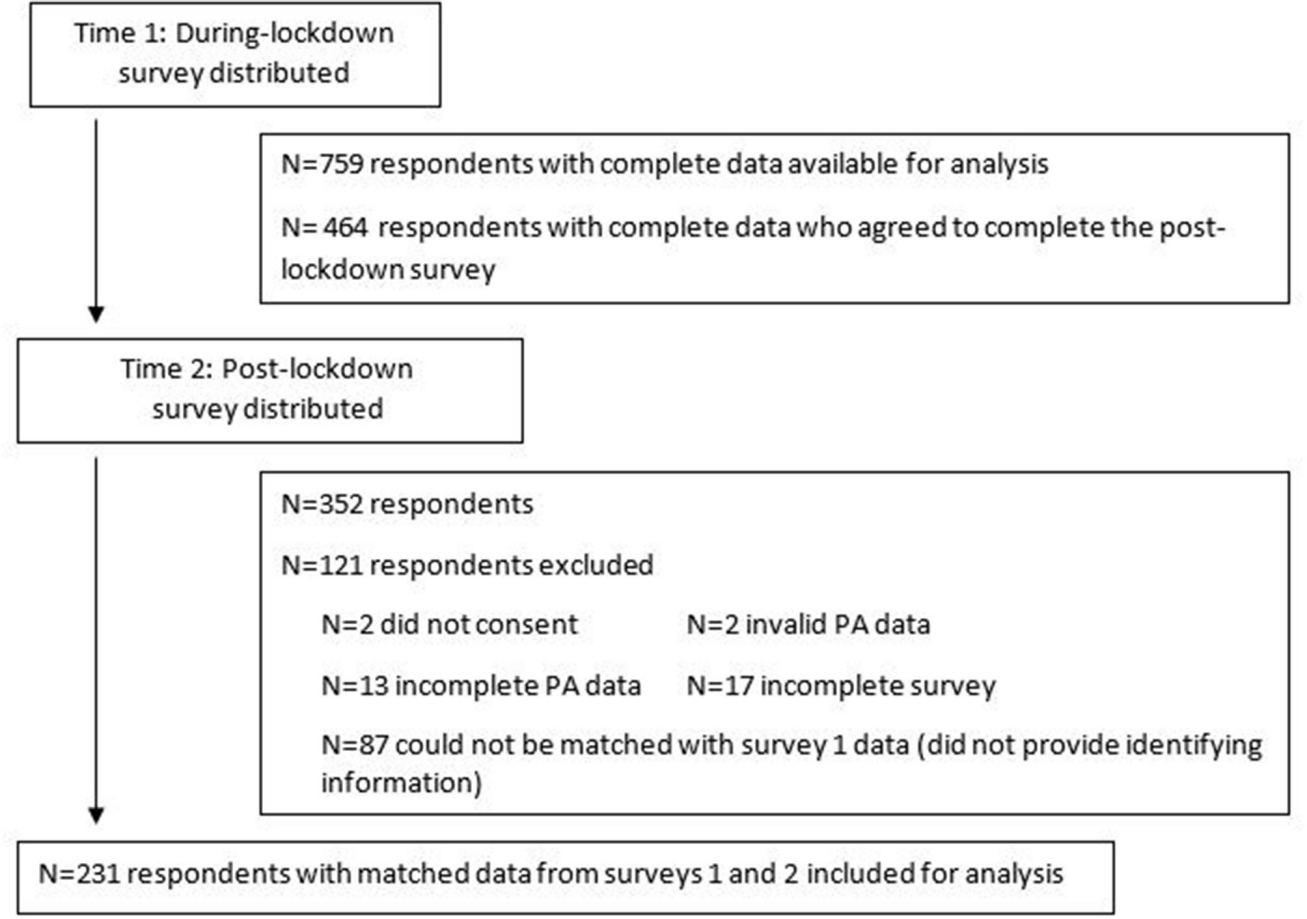
Survey responses during-lockdown (Time 1) and post-lockdown (Time 2).

The during-lockdown survey elicited 759 responses. Of those 759, 464 agreed to be contacted to complete the post-lockdown survey and 352 surveys were returned. After data cleaning (see Figure 1), the final sample for analysis consisted of 231 responses. Detailed participant characteristics of those who responded during-lockdown and the 231 with data from both surveys is shown in Table 1. Sample participants ranged in age from 18 to 81 years (mean = 42.91, SD = 13.84), were predominantly female (72.3%), NZ European (82.7%), and with at least a university degree (75.8%). Most were not essential workers (85.3%).

**Table 1:** Participant characteristics.

	**Respondents during-lockdown**	**Respondents with data from during- & post-lockdown**
Mean age (SD) years	43 (14)	43 (14)
**Gender [n (%)]**		
Female	544 (73.0)	167 (72.3)
Male	195 (26.2)	63 (27.3)
Gender diverse	3 (0.4)	1 (0.4)
Prefer not to say	3 (0.4)	0.0
**Ethnicity [n (%)]**		
NZ European	574 (82.9)	191 (82.7)
Māori	6 (0.9)	2 (0.9)
Samoan	1 (0.1)	0.0
Cook Island Māori	2 (0.3)	0.0
Chinese	4 (0.6)	0.0
Indian	2 (0.3)	1 (0.4)
Other	91 (13.2)	36 (15.6)
Prefer not to say	12 (1.7)	1 (0.4)
Essential worker (*N* = Yes, %)	102 (13.8)	34 (14.7)

### Measures

#### Physical Activity

The International Physical Activity Questionnaire-Short Form (IPAQ-SF; Craig et al., 2003) is a 7-item measure of self-reported PA, measuring the amount of moderate- and vigorous-intensity PA and walking undertaken by participants over the previous 7 days. Example items include: “During the last 7 days, on how many days did you do vigorous activities like heavy lifting, exercise classes, or fast cycling for at least 10 min at a time?” followed by the question: “How much time did you usually spend doing vigorous physical activities on one of those days?”. The IPAQ-SF has demonstrated good validity and consistency (Lee et al., 2011). For the during-lockdown survey participants completed the IPAQ-SF twice. Firstly, they were asked to report their PA from the previous week (during-lockdown). Secondly, they were asked to report their PA from a typical week prior to lockdown. To lessen the risk of recall bias we ensured that the IPAQ-SF was used in the form in which it had been validated and that it incorporated examples of moderate, and vigorous intensity activities (Matthews et al., 2012). In the post-lockdown survey, they were only asked to report their PA from the previous week (during level 2 post-lockdown restrictions).

Data screening, cleaning, and coding were undertaken according to detailed guidelines (IPAQ group, 2014). This included the truncation of data points indicating more than 960 min/week (16 h) as these are suggested to be outliers. Participants were classified as moderately, or highly active, or inactive according to proposed guidelines (IPAQ group, 2014). To be classified as moderately active, participants met one of the following criteria: (a) ≥ 3 days of vigorous intensity activity of ≥ 20 min per day; (b) ≥ 5 days of moderate intensity activity and/or walking of at least 30 min per day; or (c) ≥ 5 days of any combination of walking, moderate intensity or vigorous intensity activities achieving ≥ 600 MET min/week. The highly active participants achieved ≥ 1,500 or ≥ 3,000 MET min/week, depending on intensity of their PA and the inactive participants did not meet the criteria for moderate or high PA. PA was calculated as weekly total PA MET mins; weekly moderate intensity MET mins, weekly vigorous intensity MET mins and weekly MET mins of walking according to proposed guidelines (IPAQ group, 2014).

#### Daily Hassles

The Hassles Scale (Kanner et al., 1981) is a 117-item measure of the daily hassles participants experienced in the last 7 days. Participants were instructed to rate the degree to which each hassle item had affected their life in the past seven days (i.e., during-lockdown for the first survey administration, and post-lockdown for the second survey administration). Participants responded by selecting, “This is not a hassle for me” (0), “A somewhat severe hassle” (1), “A moderately severe hassle” (2) or “An extremely severe hassle” (3) for each item. Following Holm and Holroyd (1992), we grouped the daily hassle items according to seven categories: inner concerns (example items: troubling thoughts about one's future, trouble relaxing, concerns about inner conflicts), financial concerns (example items: financial security, concerns about job security, concerns about owing money), time pressures (example items: too many things to do, too many responsibilities, too many interruptions), work hassles (example items: job dissatisfaction, worries about decisions to change jobs), environmental hassles (example items: concerns about news events, crime), family hassles (example items: friends or relatives too far away, problems with one's children, health of a family member) and health hassles (example items: concerns about health in general, use of alcohol, concerns about weight). A severity score for each category was created by summing the item scores.

### Data Analysis

To investigate changes in PA intensity from pre- to during- and post-lockdown, in the different activity groups, a factorial repeated measures (PA intensity x time x activity group) ANOVA was conducted. To compare changes in the daily hassles categories between during-lockdown and post-lockdown, a factorial repeated measures ANOVA (daily hassles severity x time) was conducted. The Bonferroni procedure was used for *post-hoc* tests in both ANOVAs. Finally, to examine the extent to which severity of daily hassles predicted total PA during and after the lockdown, and whether any effects were moderated by activity level of participants pre-lockdown (activity group), we conducted two hierarchical multiple regressions–one using during-lockdown data and the second using post-lockdown data. In both analyses, the control variables of pre-lockdown total PA MET mins/week, age, gender, education, ethnicity, whether the respondent was an essential worker, and the number of dependent children were entered into the model first (Model 1). The severity of daily hassles during- or post-lockdown was then entered into the model as the direct predictor of total weekly PA (Model 2). Finally, the interaction between PA group and the extent of daily hassles was entered (Model 3).

## Results

Of the 231 people who provided valid PA data pre-, during-, and after-lockdown, 111 were categorized as being highly active pre-lockdown and 120 as moderately active pre-lockdown. Although 32 inactive participants completed the during-lockdown survey, they did not complete the post-lockdown survey.

### Change in PA

#### Highly Active Group (Pre-lockdown)

There was a significant main effect of the lockdown restrictions on total PA [*F*_(2,220)_ = 36.88, *p* < 0.001, partial eta^2^ = 0.40]. The follow-up Bonferroni *post-hoc* tests revealed that PA MET mins/week before lockdown were significantly higher than during-lockdown and post-lockdown. There was no significant difference between total PA during-lockdown and post-lockdown (see Table 2 for mean values).

**Table 2:** Mean (SE) physical activity pre-lockdown, during-lockdown, and post-lockdown.

	**Pre-lockdown**		**During-lockdown**		**Post-lockdown**	
	**Highly active**	**Moderately active**	**Highly active**	**Moderately active**	**Highly active**	**Moderately active**
Total PA (MET mins/week)	2,290 (40)	868 (38)	1,794 (64)	1,218 (66)	1,808 (66)	1,018 (62)
Vigorous intensity (MET mins/week)	1,285 (28)	233 (29)	951 (52)	496 (50)	936 (52)	395 (46)
Moderate intensity (MET mins/week)	543 (24)	253 (21)	419 (27)	289 (26)	433 (27)	244 (24)
Walking (MET mins/week)	462 (18)	382 (20)	424 (20)	434 (19)	439 (20)	379 (21)

There was also a significant main effect of PA intensity [*F*_(1.51,166.45)_ = 216.61, ε = 0.76, *p* < 0.001], and an interaction for PA intensity and time [*F*_(3.35,368.57)_ = 13.59, partial eta^2^ = 0.11] on total PA MET mins/week. The interaction results showed that lockdown restrictions significantly affected vigorous intensity [*F*_(2,220)_ = 30.07, *p* < 0.001, partial eta^2^ = 0.22] and moderate PA [*F*_(2,220)_ = 9.87, *p* < 0.001, partial eta^2^ = 0.08], but not walking [*F*_(2,220)_ = 1.493, *p* = 0.23]. The Bonferroni *post-hoc* tests showed that vigorous PA pre-lockdown was significantly higher than during-lockdown and post-lockdown. There was no significant difference between during- and post-lockdown. Moderate PA pre-lockdown was significantly higher than during-lockdown and post-lockdown (*M* = 432.51, SE = 26.57). There was no significant difference between during- and post-lockdown (see Table 2 for mean values).

**Figure 2:**
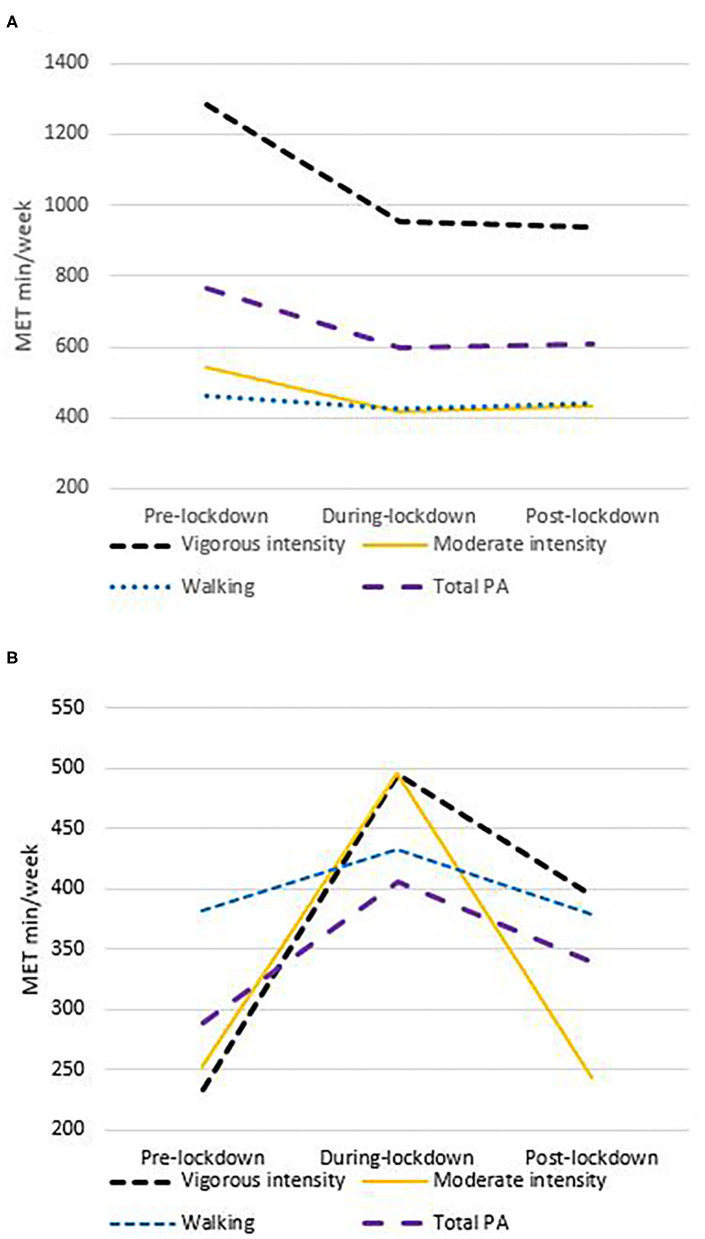
Total PA and vigorous, moderate and walking MET mins/week pre-, during and post-lockdown in highly active **(A)** and moderately active **(B)** participants pre-lockdown.

Thus, for individuals classed as highly active pre-lockdown, their vigorous and moderate intensity PA levels dropped during lockdown, and remained at this level post-lockdown. While, walking met mins/week stayed the same throughout the three time periods (see Figure 2).

#### Moderately Active Group (Pre-lockdown)

There was a significant time main effect for total PA MET mins/week [*F*_(2,238)_ = 14.92, *p* < 0.001, partial eta^2^ = 0.11]. The Bonferroni *post-hoc* tests revealed that total PA during-lockdown was significantly higher than pre-lockdown and post-lockdown. Also, post-lockdown PA was significantly higher than pre-lockdown. There was also a significant main effect for PA intensity [*F*_(1.57,187.07)_ = 11.74, ε = 0.79, *p* < 0.001], showing vigorous PA and walking were significantly higher than moderate PA (*M* = 261.92, SE = 17.59). There was no significant difference between vigorous PA and walking.

There was a significant interaction for PA intensity and time [*F*_(3.2,380.96)_ = 7.34, *p* < 0.001, partial eta^2^ = 0.06]. The results showed that vigorous PA [*F*_(2,238)_ = 14.18, *p* < 0.001, partial eta^2^ = 0.11], moderate PA [*F*_(1.41,168.21)_ = 20.82, *p* < 0.001, partial eta^2^ = 0.15] and walking [*F*_(2,238)_ = 3.54, *p* ≤ 0.03, partial eta^2^ = 0.03] were affected by the lockdown restrictions. The Bonferroni *post-hoc* tests showed that vigorous PA during-lockdown and post-lockdown was significantly higher than pre-lockdown. There was no significant difference between during- and post-lockdown. Moderate PA during-lockdown was significantly higher than pre-lockdown and post-lockdown. There was no significant difference between pre- and post-lockdown. During-lockdown walking was significantly higher than post-lockdown walking. There was no significant difference in walking between pre-lockdown and during- or post-lockdown.

Thus, for individuals classed as moderately active pre-lockdown, their participation in vigorous and moderate intensity PA increased during lockdown compared to before. Post-lockdown, they maintained their vigorous intensity PA, but moderate intensity PA returned to pre-lockdown levels. While walking did not change from pre- to during-lockdown, it reduced from during- to post-lockdown (see Figure 2).

### Experience of Daily Hassles

**Figure 3:**
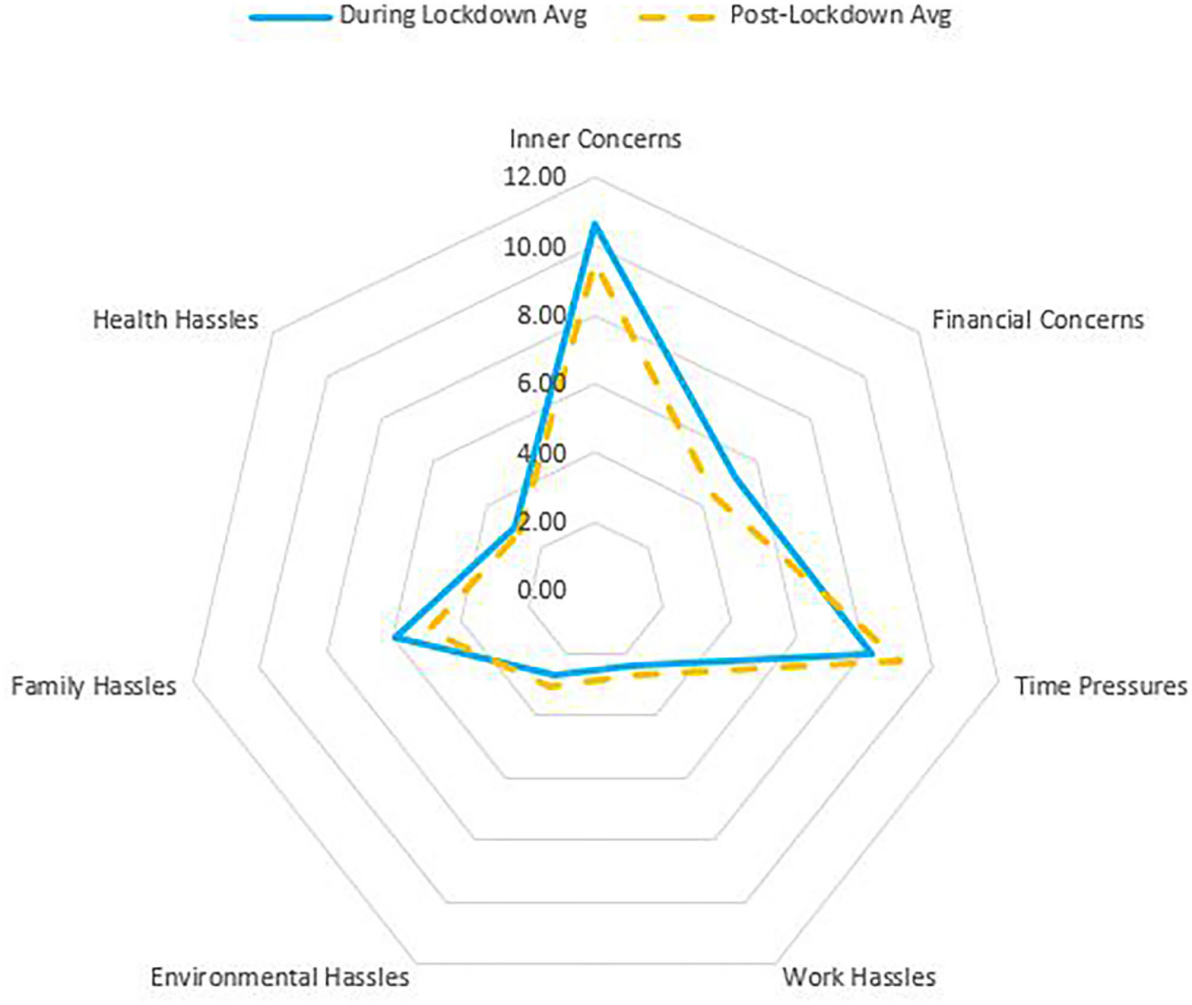
Severity of the daily hassle categories experienced during- and post-lockdown.

There were no significant differences in the severity of each daily hassle category between during- and post-lockdown (See Figure 3 and Table 3). Despite this, the scores show that the predominant daily hassles experienced during- and post-lockdown were inner concerns, time pressures, family hassles, and financial concerns.

**Table 3:** Mean (SD) severity of daily hassles experienced and percentage of sample experiencing specific hassles during and post-lockdown.

	**During lockdown**		**Post-lockdown**	
**Daily Hassle category**	**Severity**	**% Experiencing hassle**	**Severity**	**% Experiencing hassle**
Inner concerns	10.6 (9.6)	35.5	9.6 (8.9)	31.0
Time pressures	8.2 (7.2)	33.7	9.1 (7.5)	38.1
Family hassles	6.0 (4.5)	27.8	5.0 (4.2)	24.8
Financial concerns	5.2 (7.0)	18.4	4.4 (6.5)	16.7
Health hassles	3.0 (3.4)	19.9	2.8 (3.4)	19.2
Environmental hassles	2.7 (2.9)	18.3	3.1 (3.2)	20.4
Work hassles	2.4 (3.4)	17.6	2.7 (3.4)	20.3

*Higher numbers indicate higher severity of daily hassle category*.

#### Relationship Between Daily Hassles and Physical Activity During-Lockdown

Model 1, with only the control variables as predictors, explained 22% of the variance in PA behavior during-lockdown [*F*_(7,223)_ = 8.97, *p* < 0.0001]. Model 2, with the extent of daily hassles during-lockdown included as a predictor, explained 22.3% of the variance in PA behavior during-lockdown [*F*_(8,222)_ = 7.98, *p* < 0.001]. This represented a non-significant improvement of 0.3% over Model 1 [*R*^2^ Δ: *F*_(1,222)_ = 1.06, *p* < 0.30]. Model 3, with the interaction term between the extent of daily hassles during lockdown and PA group included as a predictor, did not make any improvements over Model 3 (see Table 4).

**Table 4:** Multiple regression results investigating relationships between daily hassles and PA behavior during-lockdown.

	**Model 1**				**Model 2**				**Model 3**			
	**Unstandardized β**	**Standardized β**	**Sig**.	**VIF**	**Unstandardized β**	**Standardized β**	**Sig**.	**VIF**	**Unstandardized β**	**Standardized β**	**Sig**.	**VIF**
(Constant)	994.34		0.008		1, 081.09		0.005		1, 081.20		0.006	
Pre-lockdown total weekly PA	0.41	0.44	0.00	1.09	0.40	0.44	0.00	1.10	0.40	0.44	0.00	1.99
Age	−3.14	−0.06	0.34	1.03	−3.25	−0.06	0.32	1.03	−3.25	−0.06	0.33	1.04
Gender	84.56	0.05	0.40	1.05	81.54	0.05	0.42	1.05	81.55	0.05	0.42	1.05
Education	7.45	0.02	0.74	1.03	6.74	0.02	0.77	1.03	6.74	0.02	0.77	1.03
Ethnicity	−23.52	−0.08	0.21	1.03	−23.02	−0.07	0.22	1.03	−23.02	−0.07	0.23	1.05
Essential worker	−46.29	−0.02	0.72	1.02	−47.61	−0.02	0.71	1.03	−47.63	−0.02	0.71	1.03
Children	−32.68	−0.04	0.47	1.04	−33.15	−0.05	0.46	1.04	−33.15	−0.05	0.46	1.04
Extent of daily hassles during lockdown					−1.52	−0.06	0.30	1.01	−1.52	−0.06	0.81	19.07
Extent of daily hassles during lockdown*PA group									0.004	0.05	1.00	19.25

*Dependent variable: total weekly PA during lockdown*.

#### Relationship Between Daily Hassles and Physical Activity Post-lockdown

Model 1, with the control variables as predictors, explained 48% of the variance in PA behavior post-lockdown [*F*_(8,222)_ = 25.65, *p* < 0.001]. Model 2, with the severity of daily hassles post-lockdown as a predictor, explained 49.1% of the variance in PA behavior [*F*_(9,221)_ = 23.72, *p* < 0.001]. This represented a significant improvement of 1.1% over Model 1 [*R*^2^ Δ: *F*_(1,221)_ = 4.77, *p* < 0.05]. Model 3, which included the interaction term between the severity of daily hassles and PA group as predictors did not make any improvements over Model 3 (see Table 5).

**Table 5:** Multiple regression results investigating relationships between daily hassles and PA behavior post-lockdown.

	**Model 1**				**Model 2**				**Model 3**			
	**Unstandardized β **	**Standardized β **	**Sig**.	**VIF**	**Unstandardized β**	**Standardized β**	**Sig**.	**VIF**	**Unstandardized β**	**Standardized β**	**Sig**.	**VIF**
(Constant)	348.64		0.28		506.71		0.13		496.23		0.15	2.51
Pre-lockdown total weekly PA	0.38	0.39	0.00	1.34	0.38	0.39	0.00	1.34	0.38	0.41	0.00	2.42
During lockdown total weekly PA	0.42	0.40	0.00	1.28	0.41	0.40	0.00	1.29	0.41	0.39	0.00	1.29
Age	1.19	0.02	0.67	1.03	0.22	0.00	0.94	1.06	0.22	0.00	0.94	1.06
Gender	−161.14	−0.09	0.06	1.05	−153.22	−0.09	0.08	1.05	−152.55	−0.09	0.08	1.05
Education	28.72	0.07	0.14	1.03	25.24	0.06	0.19	1.03	25.20	0.06	0.19	1.03
Ethnicity	2.85	0.01	0.86	1.04	6.16	0.02	0.70	1.05	6.62	0.02	0.69	1.10
Essential worker	−107.98	−0.05	0.32	1.03	−99.96	−0.05	0.36	1.03	−100.17	−0.05	0.36	1.03
Children	51.46	0.07	0.18	1.04	47.86	0.06	0.21	1.05	48.01	0.06	0.21	1.05
Extent of daily hassles after lockdown					−2.85	−0.11	0.03	1.05	−2.15	−0.08	0.70	18.73
Extent of daily hassles after lockdown*PA group									−0.30	−0.03	0.90	19.70

*Dependent variable: total weekly PA after lockdown*.

These results indicate that post-lockdown there was a small negative association between severity of daily hassles and PA behavior, but there was no association between daily hassles and PA during-lockdown.

## Discussion

The first purpose of this study was to examine changes in total PA and PA intensity across pre-lockdown, during-lockdown, and post-lockdown COVID-19 restrictions. We aimed to examine changes in those participants classed as low, highly and moderately active pre-lockdown. The second purpose was to investigate the extent to which the experience of daily hassles influenced any change in PA behavior. Results showed that, for individuals who were highly active pre-lockdown, vigorous and moderate intensity PA was lower during- and post-lockdown compared to pre-lockdown, while walking behavior did not change. This meant that overall, compared to pre-lockdown, total PA was lower during- and post-lockdown in highly active individuals. Although, it is important to note that, despite PA reducing as a result of lockdown, on average these individuals were still exceeding recommended PA guidelines (World Health Organization, 2010). In comparison, for individuals who were moderately active pre-lockdown, overall PA, vigorous and moderate intensity PA was higher during-lockdown compared to pre-lockdown. Post-lockdown those increases in vigorous intensity PA remained while moderate intensity returned to pre-lockdown levels. Walking behavior was higher during- compared to post-lockdown. The results also showed that the severity of daily hassles was similar during- and post-lockdown. While daily hassles had a small, but significant association with PA behavior after lockdown, there was no association during lockdown.

Our PA results show the importance of analyzing PA data collected during COVID-19 restrictions according to how active people were prior to lockdown because the pattern of change differed between the highly active and moderately active groups. Previous COVID-related research reported decreased vigorous intensity PA (Cheval et al., 2020) and moderate-vigorous intensity PA (Di Sebastiano et al., 2020) during lockdowns, but did not account for changes across groups with different levels of pre-lockdown PA. Research which did account for pre-lockdown activity level found decreases in total PA (Barkley et al., 2020) and vigorous PA (Cheval et al., 2020) in highly active participants during-lockdown. Our results support these findings but also showed lockdown resulted in lower levels of moderate intensity PA than they would normally engage in (i.e., pre-lockdown). Reductions in PA may have been due to highly active individuals being unable to perform their preferred PA as a result of the lockdown restrictions. For example, gyms were closed, sport was canceled and people were confined to exercising close to home, which restricted people from long distance cycling or running. Not being able to do their preferred PA was reported by 45% respondents in our study when they were asked to explain why PA behavior had decreased (data available on request) and has been reported in other research with highly active individuals (e.g., Constandt et al., 2020; Kaur et al., 2020). Additionally, interviews with gym-goers in India, found the closure of gyms and parks resulted in a lack of “fitness motivation” and a need to find alternatives to their usual exercise routines (Kaur et al., 2020). The fact that PA levels of highly active individuals did not revert back to “normal” levels post-lockdown (even though exercise facilities were open and sport had resumed) could be attributed to the formation of new exercise habits during lockdown (Gardner and Rebar, 2019). Our second survey took place 6 weeks after lockdown ended and importantly, took place during the winter months (the pre-lockdown survey was administered during autumn). It may be that it takes longer than 6 weeks for individuals to readjust their behavior back to normal pre-lockdown PA levels or indeed, these highly active individuals have lower PA levels (different PA habits) during the winter months compared to autumn when the pre-lockdown behavior was assessed. Assessing PA again in autumn 2021 with no lockdown restrictions in place will provide data through which to evaluate the return to pre-lockdown PA behavior controlling for seasonal variations. An alternative explanation may be that, COVID-related changes in life situations (e.g., resumption of daily commute to work; changes in workplace or employment status; caregiving for children or family members) from pre- to post-lockdown presented challenges to accumulating the previous large amounts of PA. In support of this explanation, having less time available for PA was reported by 57% of our respondents as an explanation for why their PA changed from during- to post-lockdown. Inevitably, there will be a multitude of interconnected factors that explain PA changes during and after COVID-19 restrictions. Further research, employing qualitative methods, is needed to explore the key factors and interrelationships underpinning the changes.

Participants who were moderately active pre-lockdown increased their vigorous and moderate intensity PA levels during-lockdown, and importantly, maintained vigorous PA post-lockdown. Consequently, total PA was higher post-lockdown compared to pre-lockdown. For moderately active participants, it appears lockdown provided an opportunity to increase the intensity of their pre-lockdown levels of PA. Explanations for these changes are speculative but it is possible that individuals wanted to use the time to “escape” from their homes and individuals knew being physically active was a permitted activity during-lockdown. Indeed, 35% of our participants who increased or maintained their PA levels during lockdown noted that being active “was a good excuse or reason to get outside”. Participants may also have wanted to increase their fitness, and so chose to increase at a higher intensity to achieve that. Alternatively, moderately active individuals, compared to highly active individuals, may not have needed access to specialized facilities to significantly increase their PA levels. Being active close to home during-lockdown via simple modes of PA such as running and cycling, or at home using exercise equipment and online exercise videos [which was common during lockdown (Ding et al., 2020)], may have been attractive for these moderately active individuals. Although speculative, post-lockdown, participants may have strengthened their autonomous motivation for PA (participating out of enjoyment, or the personal value attached), as a result of seeing the benefits and value of increasing their PA levels. Autonomous motivation is a strong predictor of PA behavior (Teixeira et al., 2012). Some evidence for this explanation was provided by participants who, in answer to the question “why have you been more active/continued to be active from during- to post-lockdown,” reported that it was important for physical and psychological well-being. Other researchers have pointed to the importance of supporting motivation to enable PA behavior change during the pandemic (Hudson and Sprow, 2020; Matias et al., 2020). Given that the majority of research published so far examining PA during COVID-19 restrictions has been quantitative and largely descriptive, the explanations for why PA has changed have not been thoroughly explored, but clearly would be insightful. In particular, future research to understand changes in the different types of motivation people held for PA during the periods of COVID-19 restrictions would be of value.

This study also examined whether daily hassles might help to explain changes in PA behavior. The daily hassles (i.e., stressors) a specific life event creates in a person's life has been shown to influence lifestyle behaviors (Kanner et al., 1981; O'Connor et al., 2009; Uijtdewilligen et al., 2014). We found that inner concerns, time pressures, family hassles and financial concerns were experienced most severely and were endorsed by the greatest proportion of participants both during- and post-lockdown. Contrary to hypotheses, the severity of daily hassles was not associated with PA during-lockdown. However, daily hassles had a small negative (Standardized β = −0.11) predictive effect on PA post-lockdown, which was not moderated by the participants' pre-lockdown PA status. Previous research has shown daily hassles to explain a similar proportion of variance in unhealthy snacking (Conner et al., 1999; O'Connor et al., 2009), smoking, fruit and vegetable consumption (O'Connor et al., 2009) and physical activity (Nguyen-Michel et al., 2006; O'Connor et al., 2009). While, feelings of worry/stress have been stated as reasons for decreasing engagement in positive health behaviors during lockdown (Knell et al., 2020), and those who reported decreases in PA during lockdown were more likely to report higher levels of stress (Stanton et al., 2020). It is unclear why daily hassles only predicted post-lockdown PA, even though the severity of daily hassles did not change from during- to post-lockdown. It may be that the specific daily hassles encountered post-lockdown, compared to during-lockdown (e.g., returning to work, job insecurity), presented more of a challenge to PA levels. Investigating the relationships between daily hassles and the actual stress that resulted from those hassles may explain these findings.

This study is not without its limitations. We were not able to recruit many low active participants to complete the survey. The during-lockdown survey only garnered 32 responses from participants not meeting the PA guidelines, but they either did not opt to be contacted post-lockdown or did not respond to the post-lockdown survey and so could not be included in the analysis. Therefore, we have no data on how inactive people responded to the COVID-19 restrictions. Additionally, we had a low response rate to our post-lockdown survey which severely reduced our sample size for the longitudinal analysis, this presents a significant limitation. These selection biases have resulted in a sample with a specific demographic profile (predominately of NZ European ethnicity, female and highly educated) thus limiting generalisability of our findings. Our PA data is self-report, and for pre-lockdown behavior is retrospective, which comes with clear limitations in terms of recall bias, but is consistent with the methods of other research and indeed, the only method available under these circumstances. Despite these limitations, a strength of this research is the use of the validated IPAQ-SF as the self-report measure of PA and the longitudinal nature of the data.

In conclusion, contrary to what was predicted at the outset of the COVID-19 restrictions (Papaioannou et al., 2020), PA did not decline during lockdown restrictions in participants who were meeting the WHO PA guidelines prior to lockdown, in fact moderate and vigorous intensity PA increased. For those who were exceeding the guidelines prior to lockdown, PA declined but participation during lockdown was still at a high enough level to benefit health. Post-lockdown, new PA habits had been created (highly active individuals pre-lockdown were participating in less PA compared to pre-lockdown, while moderately active individuals were more active) which may have been driven by changes in life circumstances or recognition of the importance of being physically active under COVID-19 conditions. As expected, participants reported experiencing daily hassles relating to inner concerns, family, time and finances as a result of COVID-19 restrictions, however it was only post-lockdown that these hassles were negatively associated with PA. From a practical perspective, these results suggest that the information disseminated by the NZ government on the importance of remaining active during lockdown was heeded by active New Zealanders. Health promoters should publicize these positive results and encourage people to continue being active. Drawing on our daily hassles results, as New Zealand remains in a post-lockdown period, PA promotion information needs to communicate targeted strategies to overcome the daily hassles identified to provide on-going support for PA behavior. This could include emphasizing that being physically active can help reduce the experience of stress caused by daily hassles (World Health Organization, 2010; Stubbs et al., 2017). Future research should explore the potential explanations for why PA levels of active individuals were protected from the COVID-19 restrictions. Furthermore, different methodologies that specifically target low active individuals are needed to explore potential changes in, and explanations for, their PA behavior.

## Data Availability Statement

The raw data supporting the conclusions of this article will be made available by the authors, without undue reservation.

## Ethics Statement

The studies involving human participants were reviewed and approved by University of Otago, Human Ethics Committee. The patients/participants provided their written informed consent to participate in this study.

## Author Contributions

EH, MJ, and JC coordinated data collection. CL and JC cleaned and analyzed the data. EH led the manuscript writing. All authors contributed to the final submission, conceived the study, and developed the methods.

## Conflict of Interest

The authors declare that the research was conducted in the absence of any commercial or financial relationships that could be construed as a potential conflict of interest.
